# Regulatory Mechanisms of the Wnt/β-Catenin Pathway in Diabetic Cutaneous Ulcers

**DOI:** 10.3389/fphar.2018.01114

**Published:** 2018-10-17

**Authors:** Han Zhang, Xuqiang Nie, Xiujun Shi, Jiufeng Zhao, Yu Chen, Qiuyang Yao, Chengxin Sun, Jianwen Yang

**Affiliations:** ^1^College of Pharmacy, Zunyi Medical University, Zunyi, China; ^2^College of Pharmacy, Institute of Materia Medica, Army Medical University, Chongqing, China; ^3^Pharmacy Department, Affiliated Hospital of Zunyi Medical University, Zunyi, China

**Keywords:** diabetic cutaneous ulcers, diabetic foot, Wnt/β-catenin, healing, signaling pathway

## Abstract

Skin ulcers are a serious complication of diabetes. Diabetic patients suffer from vascular lesions and complications such as peripheral neuritis, peripheral vascular lesions, and collagen abnormalities, which result in skin wounds that are refractory and often develop into chronic ulcers. The healing of skin ulcers requires an inflammatory reaction, wound proliferation, remodeling regulation, and control of stem cells. Studies investigating diabetic cutaneous ulcers have focused on cellular and molecular levels. Diabetes can cause nerve and blood vessel damage, and persistent high blood sugar levels can cause systemic multisite nerve damage based on peripheral neuropathy. The long-term hyperglycemia state enables the polyol glucose metabolism pathway to be activated, increasing the accumulation of toxic substances in the vascular injured nerve tissue cells. Sustained hyperglycemia leads to dysfunction of epithelial cells, leading to a decrease in pro-angiogenic signaling and nitric oxide production. In addition, due to impaired leukocyte function in hyperglycemia, immune function is impaired and the immune response at relevant sites is insufficient, making diabetic foot more difficult to heal. The Wnt/β-catenin pathway is a highly conserved signal transduction pathway involved in a variety of biological processes, such as cell proliferation, apoptosis, and differentiation. It is considered an important pathway involved in the healing of skin wounds. This article summarizes the mechanism of action of the Wnt/β-catenin pathway involved in the inflammatory responses to diabetic ulcers, wound proliferation, wound remodeling, and stem cells. The interactions between the Wnt signal pathway and other metabolic pathways are also discussed.

Diabetes mellitus (DM) is a systemic chronic metabolic disease with elevated blood glucose levels. The DM can cause multiple complications, including diabetic nephropathy, diabetic retinopathy, and diabetic cutaneous ulcers (DCUs). The DCU is one of the most common and serious complications of diabetes, with a quarter of patients developing a foot or skin ulcer. Each year, 50–70% of patients requiring amputations due to non-traumatic injuries are patients with DM (Lin et al., 2018). The prevalence of diabetic ulcers in Asia, Europe, and Africa is 5.5, 5.1, and 7.2%, respectively (Boulton et al., 2005). Persistent high blood sugar levels in DM patients can cause systemic multisite nerve damage based on peripheral neuropathy, leading to damage of nerves and blood vessels. A long-term hyperglycemic state activates the polyol glucose metabolism pathway, causing toxic metabolites to increase in injured vascular nerve tissue cells (Brem et al., 2007). Sustained hyperglycemia results in the dysfunction of epithelial cells, leading to a decrease in pro-angiogenic signaling and nitric oxide (NO) production (Förstermann and Münzel, 2006). In addition, hyperglycemia impairs leukocyte function causing the impairment of the immune function and an insufficient immune response, creating difficulties in the healing of foot injuries and ulcers (Gary Sibbald and Woo, 2008). The DM patients have a 15 to 28% risk of developing DCU, and 50 to 70% of these patients relapse within 5 years (Ricco et al., 2013). The average prevalence of diabetic foot in China is 5.7%, which is close to the Asian level, but lower than the global average (Reiber, 1996). The DCU is mainly associated with peripheral nerve injury and arterial disease. Clinical manifestations are grouped across three categories: 1. Skin manifestations: itchy skin, dry with no sweat, cold limbs, edema or dryness, darkening of the color, pigmentation spots, and hair loss dry skin of the extremities or blisters, blood gas bubbles, erosion, ulcers, gangrene, and necrosis. 2. Paresthesia: stinging, burning, and numbness in the extremities. Feeling slow or lost. 3. Others: development of claw foot and pulse in the dorsal artery weak or absent (Zhou and Ling, 2015). These complications of DM result in high medical costs and overcrowding of clinics. Insufficient blood supply to the limbs causes DCU patients to have vascular and peripheral neuritis complications and abnormal collagen, which lead to skin wounds that are refractory and which often ulcerate.

The increasing age of China’s population has caused the prevalence of DCU to increase annually. Treatment of DCU is one of the most critical health issues that needs to be resolved urgently in clinical practice. In recent years, DCU studies have focused on treatment at the molecular level. It has been reported that in type 2 DM, nucleotide polymorphisms (SNPs) exist for TCF7L2 of the Wnt pathway (Grant et al., 2006). The Wnt signaling pathway is reported to have a role in metabolic homeostasis (Jin, 2016). Earlier studies have discussed the role of specific steps of the Wnt signaling pathway in pancreatic β-cell proliferation. In patients with DM, this production and/or release of insulin is absent (i.e., Type 1 DM) or inefficient/exhausted (i.e., Type 2 DM), and blood glucose levels are elevated without treatment. The Wnt proteins regulate the growth factors involved in many processes, such as cell proliferation, differentiation, migration, and polarity (Chevalier et al., 2017; Nusse and Clevers, 2017; Xu et al., 2017). The role of the Wnt/β-catenin pathway in the regulation of DCU, however, has not been reported in the literature. This review examines the interplay that exists between DCU and the Wnt/β-catenin pathway. Numerous reported studies have proposed possible factors affecting the healing process. Multiple signaling paths, including Wnt signaling pathways, have become the focus of research.

## The Wnt/β-Catenin Signaling Pathway

The first Wnt gene to be described, “*int-1*,” was discovered by Nusse and Varmus (1982). Mice infected with mouse mammary tumor virus (MMTV) were found to overexpress *int-1*, which is similar to MMTV gene sequences in tumors at integration sites. The wingless gene (Wg) of the *Drosophila melanogaster* is a homolog of *int-1* and both together are known as Wnt (Dawson et al., 2013). The Wnt signaling pathway has a high degree of conservation across species, with a high degree of homology across lower organisms, such as the Drosophila species, and higher organisms, such as monkeys and humans. The Wnt family consists of at least 19 genes, including Wnt-1, Wnt-3a, Wnt-10b, and Wnt-7a (Janda et al., 2017). The β-catenin is a cytoplasm/nuclear protein that is pivotal for transcription of cell adhesion factors and the Wnt pathway, promoting cell adhesion (Kretzschmar and Clevers, 2017).

**FIGURE 1:**
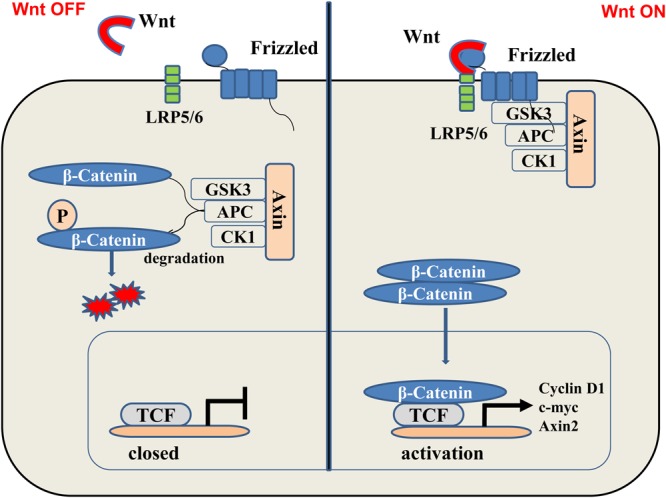
(Left) In the “off” state, β-catenin binds with glycogen synthase kinase 3β (GSK3β), axin2, adenomatous colon polyposis protein (APC), and casein kinase-1 (CK- 1). The kinase in this complex phosphorylates-catenin, thereby targeting it to the degradation of the ubiquitin proteasome system. (Right) In the “on” state, the receptor complex consisting of Frizzled and LRP5/6 binds to Wnt, which recruits Disheveled Protein (DVL) to the plasma membrane. Subsequently, several components of the β-catenin destruction complex are recruited to the membrane, which prevents the phosphorylation of β-catenin. Therefore, this protein can now accumulate in the cytoplasm and transfer to the nucleus and bind to transcription factors, thereby, stimulating the transcription of Wnt target genes such as cyclin D1, c-myc, and Axin2.

The Wnt/β-catenin pathway is one of the main processes in the regulation of wound healing and improves wound angiogenesis and epithelial remodeling. A large number of experimental studies have reported that the Wnt family is involved in many biological processes (Igota et al., 2013), including cell proliferation, apoptosis, differentiation, and the maintenance of pluripotency in stem cells (Clevers, 2006). The Wnt/β-catenin signaling can be divided into classical and non-canonical Wnt signaling. The Wnt/β-catenin signaling is activated when the Wnt ligand binds to the receptor/coreceptor (Herr and Basler, 2012). Some synthetically made Wnt proteins transfer to the Golgi apparatus, where they first bind to Wls, a transmembrane protein secreted by Wnt (van den Heuvel et al., 1993; Bänziger et al., 2006) and then are escorted from the Golgi apparatus to the cell membrane. The secreted Wnt ligand binds to the Frizzled protein (Frz) family of receptors and coreceptors, including LRP-5/6, which activates different signaling pathways (Yang H.L. et al., 2017; **Figure 1**). The interaction between the Wnt protein and its cognate receptor can be blocked by numerous soluble metabolites, including the Dickkopf protein (Dkk) or the Wnt inhibitor (WIF) (Veltri et al., 2018).

The stable conduction of Wnt/β-catenin signaling pathway is dependent on the stabilization of β-catenin. Without the Wnt signal, free cytosolic β-catenin is phosphorylated by a complex of Axin, casein kinase 1α (CK1α), and glycogen synthase kinase 3β (GSK3β). Phosphorylated β-catenin is ubiquitinated by β-transduced repetitive protein (β-TrCP) for proteasome-dependent degradation (Hurlstone and Clevers, 2002; Ahmad et al., 2017; Zhang et al., 2017). When Wnt binds to this complex, disheveled (Dvl) proteins aggregate and the expression of the degradation complex is inhibited, thus stabilizing β-catenin in cells. The β-catenin is recruited by the cytoplasm and enters the nucleus where it activates the T-cell factor/lymphoid enhancer-binding factor (LEF/TCF) protein (Yamagami et al., 2018). Interactions between β-catenin and LEF/TCF involve transcriptional regulators and histone-modification factors, which, in turn, mediate excessive developmental and homeostasis processes.

When a Wnt ligand binds to the Frz receptor and coreceptors ROR2 or RYK, β-catenin-independent Wnt signaling is triggered. These non-canonical Wnt signaling cascades are divided between Wnt/Ca^2+^ and Wnt/planar cell polarity (PCP) signaling pathways (Foulquier et al., 2018).

The Wnt/Ca^2+^ pathway, mainly activated by Wnt5a and Wnt11, causes an increase in intracellular calcium concentration and calcium activation of ion-sensitive signal components by activating G protein phospholipase C (PLC) and protein kinase C (PKC) and regulates cell motility and cell adhesion. In Wnt/Ca^2+^ signaling, Wnt ligand-receptor interactions result in the release of intracellular calcium, which acts as a secondary messenger to activate calmodulin-dependent protein kinase II (CaMKII). The CaMKII triggers the phosphorylation of transforming growth factor (TGF)-β-activated kinase 1 (TAK1), increasing the activity of lipoprotein kinase (LK), resulting in the dissociation of LEF1–β-catenin/DNA. This inhibits transcription of Wnt/β-catenin signaling activity (García-Velázquez and Arias, 2017). Simultaneously, the activated CaM induces the nuclear translocation of effectors downstream the activated T cell (NFAT) protein family, which act as a transcriptional regulators. The transcriptional activity of NFAT transcriptional is increased by mitogen-activated protein kinase p38 during Wnt stimulation (Matsumoto et al., 2017; Tan et al., 2018). In addition to the Ca^2+^-mediated signaling cascade, the induction of non-canonical Wnt signaling activates Rho-family small GTPases, including Cdc42, Rac, and RhoA, by recruiting the receptor/coreceptor/Dvl complex (Brown et al., 2017).

The PCP components interact with several signaling pathways, including the activation of the FZD/DVL PCP pathway through the PDZ and DIX moieties of the FZD/DVL complex, which induces cytoskeletal reorganization and regulates gene transcription. However, PCP signaling can also induce transcriptional responses through the c-Jun N-terminal kinase (JNK)/p38 microtubule-associated protein (MAP) kinase signaling pathway.

## The Regulatory Mechanism of the Wnt/β-Catenin Signaling Pathway in DCU

There are four overlapping processes in DCU, including hemostasis, inflammatory reaction, an increase in wound surface, and remodeling of the wound surface (Mendonça and Coutinho-Netto, 2009). The main challenges in treating DCU include a lack of new vessels in the wounds; persistent infection of the wounds; and skin dermal cell neovascularization, or poor differentiation. Lin et al. (2006) reported that upregulation of Wnt/β-catenin signaling increases the activity of high glucose-suppressed mesangial cells. The Wnt/β-catenin pathway influences the thickness and pigmentation of the skin during DCU healing (Yamaguchi et al., 2008). Effectors of the Wnt/β-catenin pathway are involved in the expression of vascular endothelial cells when glucose levels are high over a long period (Chong et al., 2007). Downregulation of the Wnt/β-catenin pathway is one of the reasons for the unpredictability of DCU, and may be due to a decrease in R-spondin 3 (Rspo-3) proteins caused by DM (Zhao et al., 2015). The Rspo family of proteins consists of four members (Rspo-1-4), which are closely related to the Wnt signaling pathway. It has been demonstrated that Rspo proteins can activate the Wnt signaling pathway, and some studies have suggested that Rspo proteins are a new class of signaling ligands that induce the canonical Wnt signaling pathway (Kim et al., 2006). However, although all Rspo proteins can activate the Wnt pathway, Rspo-3 and Rspo-2 are more effective than Rspo-1, and Rspo-4 is ineffective as an activator.

## The Mechanism of DCU

Previous studies have suggested that wound healing in patients with DM is reduced due to the following factors: 1. Glucose metabolism disorder and advanced glycation end products, which can interfere with the interaction between endothelial cells and leukocytes, and the inhibition of the function of mononuclear macrophages and endothelial cell proliferation (Lin et al., 2003). 2. Microvascular changes: manifest as extensive small vascular endothelial proliferation and capillary basement membrane thickening, causing a reduction in blood flow, local ischemia, hypoxia (Schramm et al., 2006). 3. The abnormal expression of matrix metalloproteinases (MMPs) before and after injury in DM patients: in the early stage of injury, insufficient MMPs cannot clear foreign bodies in the wound. In the later stages, overexpression of MMPs does not support collagen and matrix formation (Dasu et al., 2003). 4. Changes in epidermal growth factor receptors (EGFR): EGFR activation can promote mitosis or induce apoptosis, increase cell activity, promote protein secretion, induce cell differentiation and/or cell differentiation (Zhang et al., 2003). 5. Epidermal cell proliferation disorder: during wound healing, re-epithelialization of wounds depends on the proliferation of epidermal cells, etc. (Bickenbach and Grinnel, 2004). Today, pharmaceuticals targeting the earlier-mentioned mechanisms for treating DCU have not been successful. A recombinant platelet-derived growth factor (PDGF) has been shown to improve healing of DCU (Bhansali et al., 2009).

The healing of DCU wounds is a complex process, involving multiple cells and molecules. The processes are intertwined and the interaction between molecules and cells is mutually restrictive.

## The Wnt/β-Catenin Pathway and DCU Wound Inflammation

Inflammation is an important protective response that has a key role in the regeneration of damaged tissue and elimination of the trigger (exogenous organisms, dead cells, or physical stimuli). For example, in the wound-healing stage of ulcers, the wound is invaded by numerous inflammatory cells. Phagocytic cell fragments and the release of growth factors aid in the prevention of infection by microorganisms and have an important role in wound healing (Pekshev et al., 2017). Poor inflammatory response could increase harmful triggers, particularly the destruction of tissue by bacteria, while chronic unresolved inflammation can result in pathology, including DCU wounds and other types of wound healing. Self-limiting acute inflammation is crucial for an effective restorative response by the body. The DCU healing and injury repair promote problem solving by restoring barrier function. Inflammation is the first stage of wound repair, followed by organization formation and remodeling. Conventional inflammatory cytokines and Wnt factors control the cellular and molecular levels of mammalian tissue repair and regeneration.

Interferon-γ (IFN-γ) and lipopolysaccharides (LPS) can effectively stimulate inflammatory factors, which lead to the significant upregulation of Wnt5a (Yang X. et al., 2017). The relationship between macrophages and Wnt/β-catenin pathways has shown that macrophages and the Wnt/β-catenin pathway are closely related (Redente, 2017; Russell and Monga, 2017; Feng et al., 2018). Macrophage secretion produces the Wnt7b protein, one of the key proteins in DCU wound vascular remodeling. A close relationship between macrophages and the Wnt signaling pathway has been suggested, and Wnt5a may be the major regulator of macrophage phenotype (Newman and Hughes, 2012). In addition, there is a lot of evidence that macrophages and Wnt signaling pathways play a synergistic role in promoting angiogenesis and are involved in the process of wound repair. For example, Wnt signaling can promote angiogenesis when macrophage cells are regulated (Newman and Hughes, 2012). It has been reported that Wnt5a can promote macrophage production of pro-inflammatory factors, such as IL-6 and IL-8 (Rosell et al., 2009).

## The Wnt/β-Catenin Pathway and the Proliferation Period of DCU Wound

After inflammation of the DCU wound, the tissue forms a proliferative phase by secreting fibroblasts, vascular endothelial cells, and keratinocytes. Fibroblast growth factors (FGFs), epidermal growth factors (EGFs), nerve growth factors (NGFs), and PDGFs regulate the activation and proliferation of the repair cells (Tiaka et al., 2012). The formation of a large number of capillaries, collagen, and extracellular matrix secreted by fibroblasts constitute granulation tissue, and the proliferation of keratinocytes covers the wound surface to achieve repairing of the DCU wound (Liu et al., 2002).

The contribution of Wnt5a to vascular endothelial cell dysfunction in DM has been emphasized on recently. Endothelial cells from DM patients have a higher expression of Wnt5a expression compared to non-diabetic patients, which is associated with higher levels of activated JNK (Nusse et al., 2008). In DCU patients, this is associated with lower flow-mediated dilation (an indicator of endothelial function). *In vitro*, the inhibition of Wnt5a in endothelial cells of DM patients restores insulin-induced eNOS phosphorylation and NO production, mediated by JNK. This suggests that the Wnt5a/JNK signaling pathway is involved in the endothelial dysfunction of DM (Bretón-Romero et al., 2016).

Recent studies suggest that when Wnt and β-catenin are increased, epidermal cell proliferation, differentiation, and migration are enhanced, and wound healing is accelerated (Cheon et al., 2006; Fathke et al., 2006); the Wnt signaling pathway in skin keratinocytes affects post-traumatic skin thickness and pigmentation (Yamaguchi et al., 2008) and Wnt protects against vascular endothelial cell damage caused by high glucose levels. An abnormal regulation of β-catenin can promote or inhibit apoptosis (Chong et al., 2007) and the upregulation of Wnt/β-catenin signaling can increase the activity of high glucose-suppressed mesangial cells (Lin et al., 2006). High glucose levels inhibit endothelial cell migration and proliferation and angiogenesis through the P13K-Akt signaling pathway, and can also upregulate E-cadherin, an adhesion molecule. This work initially suggests that in high glucose-treated endothelial progenitor cells, the expression of β-catenin, EGFR and cyclin D1 is inhibited (Sun, 2012). Wnt3a stimulates the increase of H_2_O_2_ and endothelial-dependent vasodilatation in endothelial cells (Foulquier et al., 2018).

Regulating the movement of fibroblasts causes β-catenin to participate in the proliferative phase of wound repair and proliferation of dermal fibroblasts. Abnormal β-catenin can result in excessive fibrous tissue and scar formation (Mullin et al., 2017; Pérez-García et al., 2017). Phosphorylation of β-catenin and its accumulation in the cytoplasm, migration into the nucleus, and regulation of target gene transcription can result in the proliferation, migration, and accumulation in collagen of fibroblasts (Mullin et al., 2017). The size of the ulcer wound is closely related to the level of β-catenin protein expression. β-catenin influences wound healing indirectly by influencing TGF-β expression (Telerman et al., 2017).

The Wnt signaling pathway is a significant adult mammalian stem cell (ASC) regulator in mammalian epithelial cells and hair follicles (HFs). The Wnt signaling pathways appear to have a central role in the regeneration of stem cell activity, although further study on this is required. The Wnt target gene products, *Lgr5* and *Axin2*, have become common markers used to determine constitutive and damage activation of Wnt-driven ASCs.

## The Wnt/β-Catenin Pathway and Wound Remodeling Period

Optimal wound healing models the process of epidermal development, including wound remodeling, trauma to the epidermis, disruption of the epidermal barrier, and activation of the immune cells. The wound remodeling period is an important period in wound healing. After the early extracellular wound matrix is formed, the collagen skeleton and proteoglycan filling in the matrix create scar tension, through apoptosis and maturation of the cells.

On the 7th day after wound formation, fibroblasts show the contractile phenotype (terminal phenotype) myofibroblasts, resulting in shrinkage of the wound surface (Clark, 1993). During the late stages of tissue repair, fibroblasts enter apoptosis. Studies have shown that high glucose levels affect the proliferation of human fibroblasts through the Wnt/β-catenin signaling pathways. Activation of the Wnt/β-catenin signaling pathway promotes the proliferation of human fibroblasts.

The Wnt/β-catenin is one of the most critical activation initiation signals for HF development. During the second stage, a large number of keratinocytes begin to proliferate and form “hair buds” on the epidermis. At this time, cyclin D1 is upregulated and plays a regulatory role in the cell cycle (Xing et al., 2018).

## The Wnt/β-Catenin Pathway and Stem Cell Regulation

Skin epidermal stem cells are regularly regenerated and differentiated, providing the body with an unlimited source of cells. The re-epithelialization of wounds is dependent on the proliferation of skin stem cells (SSCs) during DCU healing. Porcine adult bone marrow mesenchymal stem cells have been used to generate skin (Liu et al., 2002). This enabled the clinical application of stem cells in wound healing and organ construction (Koo, 2017). Autologous stem cell transplantation for the treatment of lower extremity DCU has been reported (Guo et al., 2017). In Dubský (2014) has suggested that autologous stem cell implantation is a better treatment for DCU, compared to percutaneous transluminal angioplasty. Placental stem cells for the treatment of DCU have also been reported on (Fischkoff et al., 2017).

The advantage of self-sourced induced pluripotent stem cells (IPSCs) compared to other types of stem cells lies in that immune rejection can be minimized. Narazaki et al. (2008) studied the differentiation of IPSC into cardiovascular endothelial cells. Arteries, veins, and lymphatic endothelial cells were also successfully induced in the experiment. Recent studies have found that these cells derived from the transplantation of human-induced pluripotent stem cells (hIPSCs) can promote the regeneration of blood vessels and muscles (Hanna et al., 2007; Okita et al., 2007; Dimos et al., 2008). Tateishi et al. (2008) successful use of fibroblast-induced IPSCs in cultured insulin-producing islet cells has brought new hope for the treatment of diabetes. Chinese researchers have also tried to protect the lower limb ischemia-reperfusion injury by using IPSC (Wang, 2013). Studies have confirmed that IPSCs enter the injury site with blood flow, after the tail vein is injected into the acute lower limb ischemia-reperfusion injury, it quickly enters the damaged muscle tissue. It can be seen that the tail vein injection of IPSCs can selectively aggregate to the injury site, which has a certain therapeutic effect on acute lower limb ischemia-reperfusion injury. However, there are not much of experimental data on the treatment of diabetic foot using IPSCs, and further research is needed on the possibility of utilizing IPSCs in the treatment of diabetic foot (Tateishi et al., 2008).

The Wnt/β-catenin pathway has a role in the homeostasis of ASCs and is a focus in the regeneration of inflammatory tissue. Wnt signaling enhances the proliferation of epithelial cells (Karin and Clevers, 2016).

In contrast to a continuously regenerating epidermis, mature HFs progress through growth (growth phase), degeneration (degeneration phase), and rest (resting phase) cycles throughout their life. During the hair development cycle, the upper part of HF is permanent, while the lower part undergoes degeneration and is then regenerated by stem cells. Stem cells in HF can be divided into two groups: a set of outer layers located in the ridge, called the hair follicle stem cells (HFSCs), and the other is located in the secondary root below the bulge (sHG) (Estronca and Ferreira, 2017). The HFSCs originate from the upper region of the germinal cells in the hair base, which have an attenuated Wnt/β-catenin signal. Elevation of Wnt/β-catenin signaling in embryonic epidermal cells abrogates the specification of HFSCs and inhibits the expression of SC markers (Xu et al., 2015).

In the developing epidermis, Wnt/β-catenin signaling induces the expression of HF, and the constitutive attenuation of epidermal Wnt/β-catenin signaling impairs the formation of HF, but does not affect the integrity of the interfollicular epidermis (IFE) (Si et al., 2018). It is important to note that the consumption of β-catenin driven by K14Cre in the development of IFE creates excessive epidermal proliferation (Leirós et al., 2017). Axin2-labeled basal cells are the precursors of IFE. When β-catenin is depleted in these cells, the epidermal proliferation is poor (Adam et al., 2018). Differing epidermal hyperplasia could explain the difference between hair and glabrous epidermis. That is, the epidermis hyperproliferation of the hair skin may be caused by an inflammatory reaction of HF decomposition (Veltri et al., 2018). The β-catenin-null stem cells can differentiate into epidermis when transplanted (Kretzschmar and Clevers, 2017). However, the role of Wnt/β-catenin signaling for this to occur and maintenance of IFE is not yet known.

## Interactions Between the Wnt/β-Catenin Signaling Pathway and Other Signaling Pathways

**FIGURE 2:**
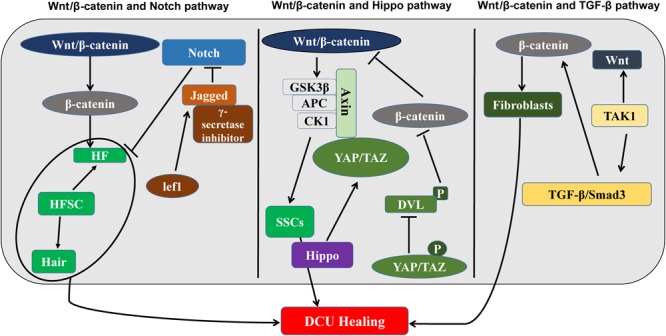
The Wnt/β-catenin signaling pathway and DCU regulatory mechanisms (left) interactions between the Wnt/β-catenin signaling pathway and the Notch signaling pathway, which regulate HF together. (Middle) Wnt/β-catenin signaling pathway and Hippo signaling interaction diagram. Regulate SSC. (Right) Interaction plots of Wnt/β-catenin signaling pathway and TGF-β signaling pathway. It is related to the production of fibroblasts. Wnt/β-catenin participates in the proliferation of fibroblasts, epidermal stem cells, and hair follicle stem cells and promotes DCU healing.

During the DCU healing process, the Wnt/β-catenin signaling pathways work independently or together with other pathways to elicit an appropriate cellular response. It is becoming increasingly clear that signaling pathways do not operate in isolation, but are strongly intertwined. Although many signaling pathways have been studied extensively, the role of some signaling pathways in DCU is unknown and requires investigation (**Figure 2**).

## The Wnt/β-Catenin Signaling Pathway and the Notch Signaling Pathway

The Wnt/β-catenin pathway cooperates with the Notch pathway, to promote DCU healing. During the DCU healing process, both Wnt/β-catenin and Notch signaling pathways are involved in the proliferative phase of wound healing, and, therefore, synergistic effects may occur in DCU regulation (Kim et al., 2017a,b). The Wnt and Notch signaling pathways regulate HF expression. Loss of Jagged1, which is the Ligand of Notch or treatment of Notch signaling with γ-secretase inhibitors can suppress new HFs in adult epidermis regulated by β-catenin. The Notch pathway is downstream of the Wnt/β-catenin signal and thus determines the destiny of HF. In addition, in cell culture, where HFSCs differentiate into hair and the *Lef1* gene is upregulated, the Wnt transcriptional target genes activate the target gene Jagged1 and directly activate Wnt/β-catenin and Notch signaling pathways (Bai et al., 2017). In contrast, in stratified IFE, β-catenin is detected in proliferating basal cells, and Notch1 is primarily expressed in the differentiated basal layer. The loss of Notch1 results in β-catenin-mediated signaling upregulation in multilayer hyperproliferating IFE, suggesting that Notch1 inhibits the Wnt/β-catenin signaling pathway to limit its activation in the basal layer (Wang et al., 2017). The synergy and antagonism between Wnt and Notch signaling appear to influence the rate of wound healing in skin.

## The Wnt/β-Catenin Signaling Pathway and the Hippo Signaling Pathway

Recently, the Hippo pathway has been reported to be connected to Wnt/β-catenin signaling. The Hippo signal transducer YAP/TAZ is part of the β-catenin disruption complex, which can coordinate the Wnt/β-catenin response regulating stem cell self-renewal and tissue homeostasis (Deng et al., 2018). In cancer cells, the downregulation of Hippo signaling is linked to the upregulation of β-catenin activity. The mechanism has been proposed as a negative correlation, where Wnt/β-catenin signaling is inhibited due to phosphorylated YAP/TAZ inhibiting the phosphorylation of Dvl and nuclear translation of β-catenin (Andl and Zhang, 2017). Due to the Hippo signal being involved in the regulation of epidermal proliferation, the interaction of the Hippo and Wnt signals could have an important role in the regulation of skin regeneration and HF regeneration and requires further investigation.

## The Wnt/β-Catenin Signaling Pathway and the TGF-β Pathways

The TGF-β and Smad signaling pathways and Wnt/β-catenin signaling pathway have some synergistic effects. Several studies have confirmed the mutual regulation of Wnt/β-catenin and TGF-β expression (Carlson et al., 2009; Kim et al., 2013; Telerman et al., 2017). The Smad3 promotes the rapid nuclear translocation of β-catenin under TGF-β stimulation in mesenchymal stem cells (Blyszczuk et al., 2016). The TGF-β-activated kinase-1 induction enables tissues to rapidly secrete Wnt proteins, promoting fibroblast differentiation (Beljaars et al., 2017). Interaction between Wnt and TGF-β follows the opposite direction expression, such as Wnt3a-induced expression of TGF-β in mouse fibroblasts is reversed (Kumawat et al., 2016).

## Conclusion and Future Research Direction

The inflammatory responses involved in the healing of DCU through the Wnt/β-catenin signaling pathways, the proliferative phase, and the remodeling phase of the wound have been discussed in this article (**Table 1**). The healing of wounds is regulated at cellular and molecular levels. Although pharmaceuticals that target the Notch and Hedgehog signaling pathways have been tested in clinical trials, pharmaceuticals regulating Wnt are currently untested in a clinical setting. The Wnt clinical trials could improve the treatment of DCU and become a new target for developing new drug treatments. The Wnt pathway interacts with the Notch signaling, Hippo and TGF-β signaling pathways. These interactions significantly increase the complexity of Wnt signaling studies, which may be a source of adverse effects in the development of pharmaceuticals. Therefore, a comprehensive approach for the preclinical phase of drug discovery is required. The role of Wnt signaling in the epidermis is diverse, due to a combination of phase and context-dependent interactions with other signal inputs. Wound healing is a complex process, and the high glucose environment of DM skin increases the complexity. For effective DCU treatment, the pathogenesis of DCU must be understood. The Wnt/β-catenin signaling pathway is involved in the wound-healing process; however, the specific mechanism is unknown. It has been reported that there is a dysfunction in the Wnt/β-catenin signaling pathway in DM refractory wounds due to a decrease in Wntl expression in high glucose environments. However, the details are unclear (Sun, 2012). It has been reported that the activation of the Wnt/β-catenin signaling pathway can promote the healing of wounds in DM rats, suggesting a new approach for the clinical treatment and development of DM refractory wounds (Sun, 2012). However, experimental drugs have adverse effects and there are limitations in their use. Future investigation into the Wnt/β-catenin signaling pathway and DM wound healing could provide a basis for diagnosis, treatment, and improved prognosis of DCU.

**Table 1:** The function of Wnt signaling in molecular biology of DCU.

Wnt pathway-associated protein	Function
Wnt3a	Promotes fibroblast proliferation
β-catenin	Promote epidermal cell proliferation, differentiation, and migration
Wnt7b	Stimulating macrophages
Wnt10b	Stimulate hair follicle development
Wnt5a	Proinflammatory factor
GSK-3β	Inhibition of apoptotic nuclear DNA
C-myc	Stimulating epidermal stem cells

## Author Contributions

HZ was responsible for the literature review and the writing. XN was responsible for corrections. XS, JZ, YC, QY, CS, and JY were responsible for proofreading.

## Conflict of Interest Statement

The authors declare that the research was conducted in the absence of any commercial or financial relationships that could be construed as a potential conflict of interest.
